# Docking data of selected human linker histone variants to the nucleosome

**DOI:** 10.1016/j.dib.2020.105580

**Published:** 2020-04-16

**Authors:** Herna de Wit, Gerrit Koorsen

**Affiliations:** University of Johannesburg, South Africa

**Keywords:** Docking, Linker histone, Nucleosome, H1, Chromatosome, Chromatin

## Abstract

Human linker histones (H1s) are important in chromatin packaging and condensation. The central globular domain of H1 anchors the protein to the nucleosome. The nucleosomal binding modes of different H1 globular domains may affect nucleosomal DNA accessibility in distinct ways. The globular domain structures of human linker histones H1.0 (GH1.0), H1.4 (GH1.4), H1t (GH1t) and H1oo (GH1oo) were homology modelled and energy minimized. A docking algorithm [validated by re-docking GH5 from the GH5-chromatosome crystal structure (PDB: 4QLC) to the nucleosome] was used to dock the modelled domains to the same nucleosome template. In addition, GH1 (PDB: 1GHC) and a protein consisting of the N-terminal and globular domains of H1x (NGH1x) were also docked using this algorithm. Models of these docked structures are presented here in the form of PDB files. The models can be used to gain more insight with regards to the nucleosomal binding modes of H1s and their individual influence on chromatin compaction.

Specifications table

**Table:** 

Subject	Biochemistry, Genetics and Molecular Biology (General), Structural Biology
Specific subject area	Molecular docking
Type of data	Table
How data were acquired	Sequences of H1 globular domains (GH1.0, GH1.4, GH1t and GH1oo) were downloaded from NCBI (https://www.ncbi.nlm.nih.gov/protein/). PDB structures of the experimentally determined globular domains (GH1, GH1x) and modeling templates were downloaded from the PDB (https://www.rcsb.org/). NGH1x was previously studied using 2D and 3D NMR (de Wit, H., Vallet, A., Brutscher, B. et al. Biomol NMR Assign (2019) 13: 249. 10.1007/s12104-019-09886-x) and modelled using TALOS+ [1,2]. MODELLER [3] was used to model GH1.0, GH1.4, GH1oo and GH1t. MD simulations were performed with GROMACS [4,5]. Molecular docking software used included MGLTools, AutoDockTools4 [6] and HADDOCK [7,8].
Data format	Raw, Analysed
Parameters for data collection	Energy scores: estimated free energy of binding; unbound system's energy; torsional free energy; final total internal energy; electrostatic energy; van der Waals, hydrogen bond and desolvation energies; final intermolecular energy.
Description of data collection	Templates for globular domain modeling were identified using a multiple sequence alignment (MUSCLE) and neighbor-joining tree. Structures of the globular domains (GH1.0, GH1.4, GH1oo and GH1t) were homology modelled to the chosen templates using MODELLER and energy minimized with GROMACS. The structures of NGH1x in low and high ionic strength conditions were modelled with TALOS+ using NMR chemical shifts obtained previously [9]. MGLTools and HADDOCK were used for docking studies of all linker histone globular domains (GH5, GH1, GH1x, GH1.0, GH1.4, GH1oo and GH1t) and NGH1x (structures from both low and high ionic strength conditions). Molecular dynamics was conducted using GROMACS.
Data source location	Institution: University of Johannesburg City/Town/Region: Auckland Park, Johannesburg Country: South Africa
Data accessibility	Data citation: de Wit, Herna; Koorsen, Gerrit (2020), “Docking data of selected human linker histone variants to the nucleosome.”, Mendeley Data, V2, doi:10.17632/tb6f5gmtbn.2
Related research article	de Wit, H., Vallet, A., Brutscher, B. et al. Biomol NMR Assign (2019) 13: 249. doi:10.1007/s12104-019-09886-x

## Value of the Data

• The data describes models of a number of chromatosome structures, which shed light on the binding modes of the globular domains of linker histones (GH5, GH1, GH1x, GH1.0, GH1.4, GH1t, GH1oo) and a version of H1x lacking the C-terminal domain (NGH1x) to the nucleosome. The data can further be used to evaluate and compare the binding modes of other linker histone variants not yet studied.
• Academic researchers in the chromatin field, structural biologists, epigeneticists, researchers in the field of drug-design and pharmacogenetics, and computational biologists can all benefit from this dataset.
• This dataset can be useful as a means to provide starting structures for MD simulations.
• This dataset can be used as a basis to develop experimental procedures, such as site-specific cross-linking, to study protein-protein or protein-DNA interactions in a nucleosome.
• Lastly, this dataset can be useful to model other linker histone globular domains in future.

## 1 Data description

Different human linker histone (H1) variants are expected to have distinct binding modes to the nucleosome.

This dataset consists of the homology models of GH1.0, GH1.4, GH1oo and GH1t, as well as the docked models of the GH1-chromatosome, GH1x-chromatosome, NGH1x-chromatosome, GH1.0-chromatosome, GH1.4-chromatosome, GH1oo-chromatosome, and GH1t-chromatosome.

The data is provided in the form of PDB coordinate files in the data repository. We also provide a PDF document in the repository providing an analysis of the data.

Table 1 gives the coordinates of the cubic box used to dock linker histone globular domains to the nucleosome.

**Table 1:** Coordinates of the cubic box used for dockings of linker histone globular domains.

Side	Coordinate	Length (*Å*)
X	28.179	126
Y	168.038	126
Z	58.343	126

Table 2 gives the genetic algorithm (GA) parameters used for the docking of linker histone globular domains to the nucleosome.

**Table 2:** Genetic algorithm (GA) parameters.

Parameter	Value
Number of GA runs	100
Population size	150
Maximum number of evaluations	2,500,000
Maximum number of generations	27,000
Maximum number of top individuals that automatically survive	1
Rate of gene mutation	0.02
Rate of crossover	0.8
GA crossover mode	twopt
Mean of Cauchy distribution for gene mutation	0.0
Variance of Cauchy distribution for gene mutation	1.0
Number of generations for picking worst individual	10

Table 3 gives the coordinates of the cubic box used to dock NGH1x to the nucleosome.

**Table 3:** Docking cubic box used for NGH1x dockings in ADT4 and HADDOCK.

Side	Coordinate	Length (*Å*)
X	28.179	110
Y	178.203	110
Z	58.343	110

Table 4 gives the energy value parameters in kcal/mol for each docked H1-chromatosome structure.

**Table 4:** Energy parameters of docked H1-chromatosome systems.

Parameter (kcal/mol)	Chromatosome								
	GH5 (docked)	GH1	GH1x	NGH1x	NGH1x	GH1.0	GH1.4	GH1t	GH1oo
Estimated free energy of binding	41.99	55.80	71.21	7.11	122.00	56.51	50.55	54.62	54.23
Unbound system's energy	0	−0.76	1.49	1.94	−4.22	−2.57	0.86	1.77	0.65
Torsional fee energy	85.02	89.79	105.00	100.50	149.45	106.79	95.16	100.23	93.07
Final total internal energy	0	−0.79	1.49	1.94	−4.22	−2.57	0.86	1.77	0.65
Electrostatic energy	−30.26	−24.04	−25.00	−25.50	−33.28	−39.16	−35.30	−36.53	−28.84
vdWaals + H-bond + desolvation energy	−12.77	−9.95	−8.79	−7.89	−4.17	−11.12	−9.31	−9.08	−10.00
Final intermolecular energy	−43.03	−33.99	−33.79	−37.39	−37.45	−50.28	−44.60	−45.61	−38.84

## 2 Experimental design, materials, and methods

### 2.1 Modeling of human linker histone globular domains

Sequences for GH1.0, GH1.1, GH1.2, GH1.3, GH1.4, GH1t and GH1oo were retrieved from NCBI's protein sequence database (https://www.ncbi.nlm.nih.gov/protein/). PDB and FASTA files of experimentally determined linker histone globular domains were also retrieved. The sequences were aligned with MUSCLE (https://www.ebi.ac.uk/Tools/msa/muscle/) [10].

Aligned sequences were imported into MEGA7 (Molecular Evolutionary Genetic Analysis) (http://www.megasoftware.net/) [11] and used to construct a Neighbor-Joining (NJ) phylogenetic tree. A distance-based method was used to construct the tree. Bootstrapping was incorporated into the tree construction [12]. Sequence similarity between the protein sequences were used to determine templates for homology modeling.

MODELLER [3,13] was used for homology modeling of GH1.0, GH1.4, GH1t and GH1oo. To select the appropriate templates for the query sequences, the alignment.compare_structures() command in the compare.py program with the BLOSUM62 matrix were used to assess structural and sequence similarities between the possible templates. A clustering tree from the input matrix of pairwise distances were created from where the template was selected.

MODELLER was used to align the query sequence with the template by taking into account structural information from the template. From the target-template alignment, a 3D model of the target was calculated using its automodel class. Five similar models based on the template structure and the alignment were generated. The best model was selected based on the lowest value of the MODELLER objective function or DOPE or SOAP assessment scores, and with the highest GA341 score.

### 2.2 Molecular dynamics for energy minimization of models

Steps of steepest descent were used to achieve a stable minimization. To avoid unnecessary distortion of the protein during the simulation, an equilibration run was done for 100 ps where all heavy atoms were restrained to their starting positions while the water was relaxed around the structure. Production runs were performed in a similar manner, except that the position restraints and pressure coupling were turned off. Production runs were performed over 10 ns on a UNIX laptop. RMSD over all backbone atoms were determined. The RMSF of each residue was calculated over the trajectory and converted to temperature factors.

### 2.3 Docking of human linker histones

Docking studies were done with MGLTools and AutoDockTools4 (ADT4) (http://mgltools.scripps.edu/) together with HADDOCK (http://haddock.science.uu.nl/). The nucleosome structure (nucleosome core + linker DNA) from the crystal structure of the GH5-chromatosome (PDB: 4QLC) was used as template for dockings. The nucleosome template was energy minimized using NOMAD-Ref [14] and force-field based normal modes calculated at 600 K to allow for optimal motions of atoms. Polar hydrogens were added and non-polar hydrogens were merged, which lead to a Gasteiger charge of −157.9602.

To validate the docking algorithm, GH5, which was originally bound to the nucleosome in the chromatosome structure (4QLC), was removed using PyMOL (https://pymol.org/2/) (GL_VERSION: 2.1 INTEL-10.25.17). GH5 was energy minimized at 600 K, non-polar hydrogens were merged, Gasteiger charges added, aromatic carbons and rotatable bonds counted, TORSDOF determined, and all guanidium residues were set to be flexible. GH5 was docked into a cubic box structure centered on the dyad-axis of the nucleosome (Table 1). Thereafter, GH5 was docked to the nucleosome using the genetic algorithm (Table 2). GH1 (PDB: 1GHC), was docked to the prepared nucleosome structure in the same manner as GH5. Interacting residues of the GH1-chromatosome were compared with the residues found to interact in the study by [15] to further validate the docking algorithm. Docked GH5- (Table 3) and GH1-chromatosomes (Table 4) agreed well with literature [15,16]. The docking algorithm was therefore applied to dock GH1.0, GH1x, GH1.4, GH1t and GH1oo to the nucleosome as described above for GH5 and GH1. NGH1x was prepared for docking and docked to the nucleosome template in the same manner as GH5, but a larger docking box was used due to the larger size of NGH1x (Table 3).

## Appendix Supplementary material

Image, application 1
